# Maximum likelihood estimation of age-specific incidence rate from prevalence

**DOI:** 10.1371/journal.pone.0321924

**Published:** 2025-05-14

**Authors:** Sabrina Voß, Annika Hoyer, Ralph Brinks

**Affiliations:** 1 Chair for Medical Biometry and Epidemiology, Faculty of Health/School of Medicine, Witten/Herdecke University, Witten, Germany; 2 Biostatistics and Medical Biometry, Medical School OWL, Bielefeld University, Bielefeld, Germany; 3 Institute for Biometry and Epidemiology, German Diabetes Center, Düsseldorf, Germany; San Giuseppe Hospital, ITALY

## Abstract

Usually, age-specific incidence rates of chronic diseases are estimated from longitudinal studies that follow participants over time and record incident cases. However, these studies can be cost- and time-expensive and are prone to loss to follow up. An alternative method allows incidence estimation based on aggregated data from (cross-sectional) prevalence and mortality studies using relations between incidence, prevalence and mortality described by the illness-death model and a related partial differential equation. Currently, adequate options for the assessment of the accuracy of the achieved incidence estimates are missing and bootstrap resampling methods are used instead. Therefore, we developed novel ways to estimate incidence rates based on the maximum likelihood principle with corresponding confidence intervals. Historical data about breathlessness in British coal miners and diabetes in Germany are used to illustrate the applicability of this method in scenarios with non-differential and differential mortality. We have two scenarios of available data in the case of differential mortality: mortality of diseased and all-cause mortality, or all-cause mortality and mortality rate ratio. Our results show that estimation of incidence rates and corresponding confidence intervals of chronic conditions based on aggregated data with the maximum likelihood method using a binomial likelihood function is possible and can replace resampling techniques.

## Introduction

Age-specific incidence rates may provide hints about disease etiology, vulnerable groups and (success of) disease prevention. They are typically estimated from longitudinal studies, for instance, cohort studies, where initially disease-free study participants are followed over time and incident cases are recorded. However, longitudinal studies can be lengthy, expensive and are prone to loss to follow up [1]. Typically, a cross-sectional design is easier to conduct with respect to duration of data collection. Cross-sectional studies allow estimation of age-specific prevalences of diseases. In case of chronic diseases, established interrelations between incidence rate and prevalence can be used to estimate the former from the later based on aggregated prevalence data. In 2016, Landwehr & Brinks [2] compared different deterministic approaches to this task. A method based on a differential equation [3] similar to the Kolmogorow Forward Equation [4] turned out to be superior with respect to mean absolute error. For conclusions of inferential statistics in the differential equation approach, we had to use re-sampling techniques so far as shown, for example, in Brinks et al. (2015) [5]. In this work, we propose a novel maximum likelihood (ML) approach and put special emphasis on estimating confidence intervals, which is mandatory in many statistical analyses [6]. We first briefly review the illness-death model for chronic conditions and mathematical relations between the age-specific prevalence and the transition rates in the illness-death model. As applications, we use a historical data set about breathlessness in British coal miners [7] and data about type 2 diabetes in Germany [8]. These data are used to obtain the age-specific incidence rates from aggregated prevalence data. In addition to that, confidence intervals for the ML-estimator of the age-specific incidence rate are estimated as well. We distinguish different situations according to the mortality rates. In the situation of non-differential mortality, people with and without the disease of interest have equal mortality rates ($m_{0}=m_{1}$). The case where the mortality rates are unequal ($m_{0}\neq m_{1}$) is called differential mortality [9]. This differential mortality is a reasonable assumption for many diseases. We will have our analysis in three different scenarios: 1) non-differential mortality; 2) differential mortality given the mortality of diseased and all-cause mortality (general mortality rate); 3) differential mortality given the general mortality and mortality rate ratio (the ratio of mortality rates of non-diseased and diseased individuals).

The aim of our work is to propose a method for the estimation of the incidence rate from prevalence and associated confidence intervals, using a maximum likelihood method based on a (partial) differential equation that links prevalence, incidence rate and mortality rates in an illness-death model for a chronic condition.

## Methods

### Data sets

The maximum likelihood method in this article is based on aggregated current status data where information on disease status is collected at one time-point (prevalence data). The usage of this method for the estimation of incidence rates and corresponding confidence intervals will be analyzed based on two different data sets. The first example investigates fictional data on breathlessness in British coal miners as the chronic condition. The second example is a real data set about type 2 diabetes in women in Germany. Data and source code in the statistical programming language R (The R Foundation for Statistical Computing) are provided in the free online repository Zenodo under DOI 10.5281/zenodo.8383573. Calculations and results were produced using R version 4.1.0 on a 64-bit Linux notebook.

### Data set 1: Breathlessness in British coal miners

The chronic condition under consideration in data set 1 is breathlessness in British coal miners. This fictitious data was published in Elandt-Johnson and Johnson (2014). Table 1 shows information on the associated prevalence data. It reports on age-specific aggregated data stratified by age groups from 20 to 64 with a size of 5 years for every group. Prevalence data are presented as the number of persons observed (*n*_k_), the number of persons with breathlessness (*c*_k_) and the age-specific prevalence for all k age groups (k = 1, …,9) [7].

**Table 1:** Fictitious example data about breathlessness in British coal miners taken from table 14.2 a in [7] stratified by age groups with numbers of persons observed, number of persons with breathlessness and age-specific prevalence for 5-year age groups from 20 to 64.

Age group *k* (in years)	Number of persons observed (*n*_*k*_)	Number of persons with condition (*c*_*k*_)	prevalence (*p*_*k*_) of breathlessness (in %)
**20 to 24**	1952	16	0.820
**25 to 29**	1791	32	1.787
**30 to 34**	2113	73	3.455
**35 to 39**	2783	169	6.073
**40 to 44**	2274	223	9.807
**45 to 49**	2393	357	14.92
**50 to 54**	2090	521	24.93
**55 to 59**	1750	558	31.89
**60 to 64**	1136	478	42.08

Besides the data presented in Table 1, we will also incorporate mortality in the maximum likelihood estimation: Table 2 shows the life tables for the general population and the population with breathlessness in Wales and England for the same age groups as the prevalence data.

**Table 2:** Fictitious example data about breathlessness in British coal miners taken from table 14.2 b in [7] with life tables for England and Wales in the general population and in the population with breathlessness stratified by 5-year age groups from 20 to 64.

	Life tables	
Age group *k* (in years)	General population	Population with breathlessness
**20 to 24**	481185	343937
**25 to 29**	478683	333343
**30 to 34**	476150	320446
**35 to 39**	472641	304305
**40 to 44**	467066	284325
**45 to 49**	457729	260806
**50 to 54**	441895	233060
**55 to 59**	415262	200561
**60 to 64**	372908	163241

The data from Table 1 will be used for the maximum likelihood estimation in case of non-differential mortality. The information in Table 2 will be additionally used in the example for differential mortality with the mortality of diseased and the general mortality given.

The general mortality in the example is given by m(a)=exp(−9.300+0.092·a) and the mortality of diseased individuals by $m_{1}(a)=exp(-6.295+0.052\cdot a)$ (see Source Code on Zenodo for explanation).

### Data set 2: Type 2 diabetes in Germany

The second example uses data about type 2 diabetes in German women in 2009 and 2010. Table 3 has aggregated data about the ascertained diagnoses of type 2 diabetes of women in the years 2009 and 2010 taken from the German statutory health insurance. A detailed description of this data can be found in [8].

**Table 3:** Numbers of observed women and women with type 2 diabetes in Germany in the years 2009 and 2010 stratified by age groups with 5 year size from 20 to 99 years. Data from the statutory health insurance.

Age group *k* (in years)	Year 2009		Year 2010	
	Number of women observed (*n*_*k*_)	Number of women with type 2 diabetes (*c*_*k*_)	Number of women observed (*n*_*k*_)	Number of women with type 2 diabetes (*c*_*k*_)
**20 to 24**	19029	33	18939	36
**25 to 29**	19549	65	18917	69
**30 to 34**	19391	109	19388	122
**35 to 39**	20885	200	19722	208
**40 to 44**	28844	402	26543	403
**45 to 49**	28856	706	29509	742
**50 to 54**	25641	1145	25870	1178
**55 to 59**	23223	1826	23238	1850
**60 to 64**	18845	2134	20112	2423
**65 to 69**	21964	3160	19714	2887
**70 to 74**	22965	4281	23452	4446
**75 to 79**	15944	3628	16509	3909
**80 to 84**	13310	3114	13083	3295
**85 to 89**	8796	2159	8637	2220
**90 to 94**	2380	569	2760	681
**95 to 99**	892	188	833	177

Due to legal restrictions in the use of the original data, random noise (2%) has been added to the original data. After this, the data has been downsampled by the factor of 100 and rounded to the nearest integer. Table 3 summarizes the data for age groups from 20 to 99 years with a size of 5 years for every age group. Presented are the numbers of women observed in the age group (*n*_k_) and the numbers of women with diagnosed type 2 diabetes (*c*_k_) for both years 2009 and 2010.

This data set is used to demonstrate the analysis in presence of differential mortality when the mortality rate ratio and the general mortality are known. Values for the general mortality *m* are taken from the German Federal Statistical Office [10]. Values for the mortality rate ratio *R* are taken from the Danish Diabetes Register [11]. This transfer is possible as rate ratios provide a stable measure of association in a wide variety of human populations [12].

Both values are used as a function in age *a*:


m(a)=exp(−11.35+0.1061·a)

$$R(a)=\max\left\{\exp\left(\log(6.5)-(a-20)\cdot \frac{\log(6.5)-\log(2)}{50}\right),1\right\}.$$

### Illness-death model

**Fig 1:**
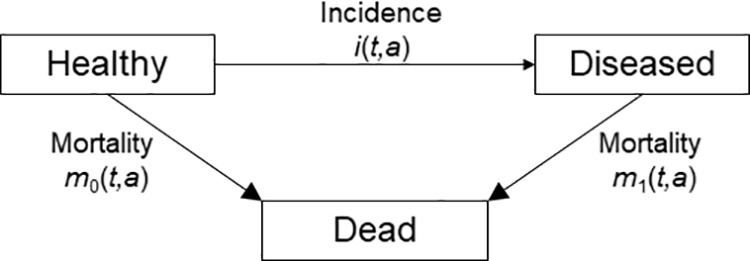
Illness-death model for a chronic condition (‘Diseased’) and associated transition rates: incidence rate i, mortality rate without (m_0_) and with the disease (m_1_).

Fig 1 shows the illness-death model (IDM). The possible transitions and associated rates are the age-specific incidence rate *i*, the mortality rate of non-diseased *m*_0_ and the mortality rate of diseased *m*_1_. In addition to that, the underlying chronic condition has the prevalence *p*. Recently, it has been shown that a partial differential equation (PDE) relates the age-specific prevalence *p* of a chronic condition at some time *t* to *i*, *m*_0_ and *m*_1_[13,14].

These measures generally depend on the calendar time *t* and on age *a*. For instance, *p*(*t, a*) denotes the fraction of people alive with the condition and *i*(*t*, *a*) is the incidence rate of the people aged *a* at time *t*, respectively [13]. In epidemiological contexts, the calendar time *t* is sometimes called period [15]. Note that we only consider chronic conditions (persistent, irreversible) so there is no transition from the *Diseased* state back to *Healthy* (Fig 1).

The PDE linking the transition rates from the IDM in Fig 1 with the age-specific prevalence *p* is given by [16]


$$i=\frac{(\partial /\partial t+\partial /\partial a)p}{\left(1-p\right)}+p\left(m_{1}-m_{0}\right).$$ (1)

After solving the formula in equation (1) for *i* this leads to

$$i=\frac{(\partial /\partial t+\partial /\partial a)p}{\left(1-p\right)}+p\left(m_{1}-m_{0}\right).$$

In some applications, the mortality rates *m*_0_ and *m*_1_ are unknown and only the general mortality *m* of the overall population and the mortality rate ratio R = *m*_1_/*m*_0_ are available. The general mortality can be expressed in terms of the prevalence and the mortality rates (and respectively R) with the following equation:


m =  (1 - p) m_{0} +  p m_{1} =  m_{0} [1 +  p (R - 1)] (2)

This relation can be used in calculations when *m*_0_ and/or *m*_1_ are unknown [16].

### Maximum likelihood estimation

Aggregated current status data is given for K age groups indexed k (k = 1,..., K). Let *c*_k_ be the number of people with the chronic condition in age group k and *n*_k_ the overall number of people in age group k.

It is assumed that the number of individuals with the chronic condition under consideration is binomially distributed. The corresponding probability mass function is given by (nkck)pkck(1−pk)nk−ck for k = 1,..., K. Then, the binomial likelihood function *L* for the aggregated current status (see Table 3 as an example) data is:


L=∏k=1K(nkck)pkck(1−pk)nk−ck (3)

with *p*_k_ as the age-group-specific prevalence [17]. When the prevalences *p*_k_ from Equation (3) have an analytical representation as a function of the rates *i*, *m*_0_, and *m*_1_ (for example: *p = p(i, m*_0_, *m*_1_)), the estimation of parameters can be straightforward. This will be demonstrated with data about breathlessness in British coal miners. Aim of this concept is the substitution of the prevalence with a functional relation based on the relations in equation (2) and estimation with the maximum likelihood method. Prevalence (and if needed also mortality rates) are estimated with the maximum likelihood method with given prevalence and mortality data. This will result in a plug-in-ML-estimator of the incidence rate using the relations between the rates described with the PDE. The mortality rates and the mortality data given determine the resulting partial differential equation. Consequently, the applicability of the method depends on the mortality rates and the mortality data.

Therefore, we perform our approach in three different scenarios with non-differential and differential mortality in diseased and non-diseased individuals (based on the data sets described above). In the case of differential mortality, the type of mortality data that is available is of importance. For example, it is possible that the mortality rate *m*_1_ of diseased and the general mortality *m* in the overall population are available or that only the general mortality *m* and the mortality rate ratio *R = m*_1_/*m*_0_ are available. The second case is the epidemiologically more relevant case since this case occurs more frequently in reality.

### Likelihood in case of non-differential mortality

In our first example we assume non-differential mortality. Persons without and with the chronic condition (breathlessness in the example) have equal mortality rates ($m_{0}=m_{1}$). The non-differential mortality reduces equation (1) to


$$\left(\frac{\partial }{\partial t}+\frac{\partial }{\partial a}\right)p=(1-p)i.$$ (4)

With the assumption that the prevalence is independent of time *t* and only depends on age *a,* the PDE in equation (1) becomes the following ordinary differential equation (ODE) in *a*:


$$\partial_{a}p:=\frac{\partial p}{\partial a}=(1-p)i.$$ (5)

Equation (5) has the general solution in (6) with the initial prevalence $p_{0}=p(a_{0})$:


$$p(a)=1-(1-p_{0})\exp\left(-\int_{a_{0}}^{a}i(\tau )d\tau \right)$$ (6)

(See formula (12) in [16]). Equation (5) is the basis for a straightforward estimator of the incidence rate as i(a) can be written as $\frac{\partial_{a}p}{\left(1-p\right)}$. We make the approach to write the incidence rate as: i(a= exp$\left(\beta_{0}+\beta_{1}a\right)$ (with $\beta_{0},$ $\beta_{1}$ as coefficients, see also results section). The substitution of this formula in equation (6) using the initial age 20 and the initial prevalence $p_{0}=p(a_{0}=p(20)=0$ gives: p(a=1− exp(h(20)−h(a)) with the auxiliary function h(z)= $\frac{\exp(\gamma_{0}+\gamma_{1}z)}{\gamma_{1}}$. Details about the analytical steps are provided in a supplementary file. With these evaluations, the Likelihood function in equation (3) is given by L(γ0,γ1)=∏k=1K(nkck)p(γ0,γ1)kck(1−p(γ0,γ1)k)nk−ck with ${p(\gamma_{0},\gamma_{1})}_{k}=1-$ exp$\left(\frac{\exp(\gamma_{0}+\gamma_{1}20)}{\gamma_{1}}-\frac{\exp(\gamma_{0}+\gamma_{1}k)}{\gamma_{1}}\right)$.

### Likelihood in case of differential mortality

In case of differential mortality, we have to distinguish the situations depending on which type of mortality data is given. In our first example (the data about the coal miners), the mortality rate *m*_1_ of the diseased and the general mortality *m* in the overall population are available. In the second example (diabetes in German women in 2009 and 2010), we consider the case where the general mortality m and the mortality rate ratio $R=m_{1}/m_{0}$ are available.

### Mortality of diseased and general mortality

We assume the case that we have differential mortality in the data about British coal miners with the mortality of diseased and the general mortality given. Starting with the PDE in equation (1) and using the information from equation (2) that says that the general mortality rate (m) is a convex combination of the mortality rate of non-diseased ($m_{0}$) and the mortality rate of diseased ($m_{1}$) we obtain the following PDE [16]:


$$\left(\frac{\partial }{\partial t}+\frac{\partial }{\partial a}\right)p=i-p\left({i+m}_{1}-m\right).$$ (7)

With the assumption of independence from time t this reduces to an ODE:


$$\partial_{a}p=i-p({i+m}_{1}-m)$$ (8)

The general solution of this ODE is given by:


$$p(a)=\exp\left(-G(a)\right)\{p_{0}+\int_{a_{0}}^{a}i(\tau )\exp(G(\tau ))d\tau$$ (9)

In equation (9) the function G(a) is given by G(a)=$\int_{a_{0}}^{a}\{i+m_{1}-m\}(\tau )d\tau$ and $a_{0}$ is the initial condition with $p_{0}=p(a_{0})$ [18]. 

In accordance to the example with non-differential mortality we assume that the incidence rate can be calculated with i(a= exp$\left(\gamma_{0}+\gamma_{1}a\right)$ with the coefficients $\gamma_{0}$ and $\gamma_{1}$. This incidence rate is then substituted into equation (9) with the initial condition $a_{0}=20$ and $p(20)=p_{0}=0$.

After inserting the prevalence in equation (3), the likelihood is given by


L(γ0,γ1)=∏k=1K(nkck)p(γ0,γ1)kck(1−p(γ0,γ1)k)nk−ckwith

$${p(\gamma_{0},\gamma_{1})}_{k}=\exp\left(-G(k)\right)\{\int_{20}^{k}\exp(\gamma_{0}+\gamma_{1}\tau )\exp(G(\tau ))d\tau \}$$

### General mortality and mortality rate ratio

The third scenario shows calculations in the case of differential mortality with general mortality and mortality rate ratio given (example with diabetes in German women in 2009 and 2010 from Table 3). With $m=(1-p)m_{0}+{pm}_{1}$ and $R=\frac{m_{1}}{m_{0}}$ the PDE in equation (1) is


$$\left(\frac{\partial }{\partial t}+\frac{\partial }{\partial a}\right)p=(1-p)\left\{i-m\frac{p(R-1)}{1+p(R-1)}\right\}$$ (10)

and can be solved for the incidence rate with:


$$i=\frac{(\partial /\partial t+\partial /\partial a)p}{1-p}+m\frac{p(R-1)}{1+p(R-1)}.$$ (11)

Given the data from Table 3 we make the following approach for the prevalence p(t,a) = expit $\left(\beta_{0}+\beta_{1}t+\beta_{2}a+\beta_{3}a^{2}\right)$ with dependence on two time scales: calendar-time t and age a. We then calculate the maximum likelihood estimator for the coefficients $\beta_{0},\beta_{1}$, $\beta_{2}$, $\beta_{3}$ and the Likelihood function


L(β0,β1,β2,β3,t)=∏k∈{22.5,…,97.5}(nkck)p(t,a)ck(1−p(t,a))nk−ck

with t∈{2009,2010}. As this was a nonlinear optimization problem, we solved it with the BFGS method and the initial values -2.3, 0.1, 0 and -0.001. After the maximum likelihood estimation of p, we can find the estimate for the incidence rate *i* with equation (11) using the plug-in method (non-parametric method for the estimation of functionals) where the incidence rate is used as a statistical functional in p. The plug-in method then offers the opportunity to estimate a Normal-based interval for the incidence rate (more information about this method are in [17]). The calculation of confidence intervals for *i* is done with the delta method. The delta method estimates the standard errors of i with a transformation of the standard errors in p using a differentiable function (g) that transforms p to i and its derivative for the calculation of the variance of i [17] (see results section). All upper and lower bounds of the confidence intervals were calculated based on the Fisher information matrix and subsequent asymptotic normal approximation [19].

## Results

In presenting our results, we will first show the estimation in the case of non-differential mortality using the data in Table 1 from the example about breathlessness in coal miners. The second example is in the presence of differential mortality with the mortality of diseased and the general mortality given by usage of the data in Table 2 (coal miners). The third approach has differential mortality with the mortality rate ratio and the general mortality known.

### Non-differential mortality

In our first example we assume non-differential mortality where persons without and with breathlessness have equal mortality rates ($m_{0}=m_{1}$). A linear regression model was fit to logit(p(a)) with


$$logit\left(p(a)\right)=log\left(\frac{p(a)}{1-p(a)}\right)=\beta_{0}+\beta_{1}a.$$

and the midpoints of the age groups in Table 1 as the ages for evaluation (a = 22.5, 27.5, …, 62.5). Using the prevalences in Table 1 for fitting the model we get $\beta_{0}$= -7.02 and $\beta_{1}$= 0.11.

We use the following aspects to get the corresponding age-specific incidence rate:

1. The expit-function is the inverse of the logit function with expit = logit^-1^ = $\frac{\exp}{1+\exp}$
2. The derivate of expit is expit∙(1–expit)
3. p(a= expit $\left(\beta_{0}+\beta_{1}a\right)$
4. $p^{\prime }(a)=$ $\beta_{1}$ [1− expit$\left(\beta_{0}+\beta_{1}a\right)$]∙ $expit\left(\beta_{0}+\beta_{1}a\right)$ = $\beta_{1}\left(1-p(a)\right)\cdot p(a)$

With these and equation (5) we can rewrite the age-specific incidence rate as: $i(a=\frac{\beta_{1}\left(1-p(a)\right)\cdot p(a)}{\left(1-p(a)\right)}=\beta_{1}\cdot p(a)$ = $\beta_{1}$∙$expit\left(\beta_{0}+\beta_{1}a\right)$.

**Fig 2:**
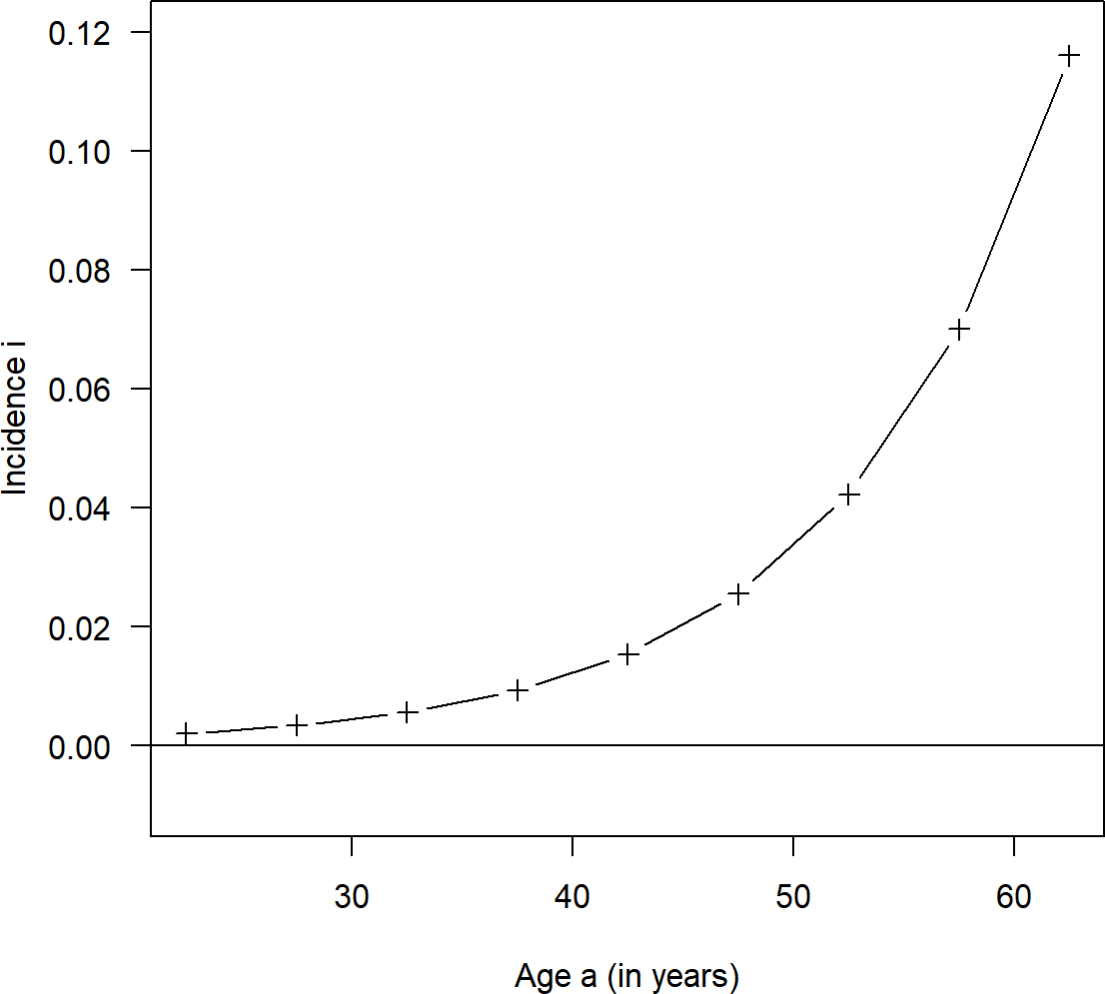
Estimated incidence rates (with this functional relation) for the midpoints of the age groups as a black line.

With $i(a)=\beta_{1}\cdot p(a)$ = $\beta_{1}$∙ $expit\left(\beta_{0}+\beta_{1}a\right)$ and data from Table 1 we get the (ML-estimated) incidence rate for the example about breathlessness in British coal miners with: i(a=0.11∙expit(−7.02+0.11a). Fig 2 shows the estimated incidence rates (with this functional relation) for the midpoints of the age groups as a black line.

It also shows that the age-specific incidence rate *i(a)* of breathlessness grows exponentially with age. The maximum likelihood estimation of the prevalence given by p(a=1− exp(h(20)−h(a)) with the auxiliary function h(z)= $\frac{\exp(\gamma_{0}+\gamma_{1}z)}{\gamma_{1}}$ (with the initial age 20 and the initial prevalence $p_{0}=0$) is evaluated at the same midpoints of age groups as before (a = 22.5, 27.5,…, 62.5) and substituted into equation (3) to get a likelihood function ($L(\gamma_{0},\gamma_{1})$) that was used to obtain the maximum likelihood estimator for the coefficients $\gamma_{0}$ and $\gamma_{1}$. The point estimates were $\gamma_{0}=-7.823$ and $\gamma_{1}=0.0756$ leading to i(a= exp(−7.823+0.0756·a) for the calculation of age-specific maximum likelihood estimates of the incidence rate. Additionally, the 95% confidence intervals for the parameters are estimated using the inverse of the Fisher information matrix for large sample approximation of the variance-covariance matrix [19]. Table 4 shows the point estimates and the resulting 95%-CI for $\gamma_{0}$ and $\gamma_{1}$.

**Table 4:** Maximum likelihood estimators for the coefficients γ_0_ and γ_1_ used for parameterization of the age-specific incidence rate of breathlessness in British coal miners without differential mortality.

	Point estimate	95% confidence interval
γ_0_	-7.823	-8.058 to -7.588
γ_1_	0.0756	0.0701 to 0.0811

### Differential mortality

In the case of differential mortality, we distinguish two situations with either the mortality rate *m*_1_ of the diseased and the general mortality *m* in the overall population (the data about the coal miners) or the general mortality m and the mortality rate ratio $R=m_{1}/m_{0}$ given (Diabetes in German women in 2009 and 2010).

### Mortality of diseased and general mortality

In the data about breathlessness in British coal miners we assume to have differential mortality with the mortality of diseased and the general mortality as the aggregated mortality info given in this case. Table 2 has the information that we use for the estimation of the mortality rate in the general population of England and Wales as well as the mortality rate of British coal miners with breathlessness. This is done with the theory of single decrement processes. More information about this can be found in Chapter 3 of [20]. The 5-year life tables in column 2 and 3 are converted to one-year probabilities of dying at first.

Table 2 is used to estimate the mortality rate of British coal miners and the general mortality in England and Wales.

The following age groups are used: 20–24, 25–29, 30–34, 35–39, 40–44, 45–49, 50–54, 55–59, 60–64.

Column 2 in Table 2 has the Life tables of the General population ($S_{x}$) and Table 2 column 3 has the Life tables of the population with breathlessness ($S_{xp}$).

These data that is given in 5-year steps are used for the calculation of the 1-year probability ($p_{mk}$) in the first step with the following equation:


$$p_{m_{k}}=1-{\left(1-\frac{S_{x_{k}}-S_{x_{k+1}}}{S_{x_{k}}}\right)}^{0.2}$$

Here the Life tables from the next elder age (k+1) are substracted from age group k. A linear regression then models these one-year probabilities via log($p_{m})=\beta_{0}+\beta_{1}a$ with a as age.

The coefficients estimated from these linear regression models are used to get the mortality rates. Table 5 shows the estimated coefficients.

**Table 5:** Estimated coefficients and mortality rates in breathlessness example in case of differential mortality.

	Population with breathlessness	General population
$\hat{\beta_{0}}$	−6.295098	−9.300141
$\hat{\beta_{1}}$	0.0529716	0.09189776
mortality rate	$m_{1}(a)=$ −6.295098+0.0529716·a	$m_{1}(a)=$ −9.300141+0.09189776·a

As the mortality rates are unknown, these probabilities are modeled in a linear regression model and the resulting coefficients are then used to define the mortality rates $m_{1}(a)$ and m(a). A more detailed description of these calculations as well as the resulting mortality rates can be found in the supplementary document.

Inserting i(a= exp$\left(\gamma_{0}+\gamma_{1}a\right)$ into equation (9) with the initial conditions $a_{0}=20$ for age and $p(20)=p_{0}=0$ for the starting prevalence the maximum likelihood estimator with 95% confidence intervals for $\gamma_{0}$ and $\gamma_{1}$ in Table 6 are calculated.

**Table 6:** Maximum likelihood estimators for the coefficients γ_0_ and γ_1_ used for parameterization of the age-specific incidence rate of breathlessness in British coal miners with differential mortality.

	Point estimate	95% confidence intervals
γ_0_	-8.471	-8.7296 to -8.2116
γ_1_	0.1010	0.09480 to 0.1073

### General mortality and mortality rate ratio

In the example with data about diabetes in German women in 2009 and 2010 shown in Table 3 the general mortality m and the mortality rate ratio R are known (see Methods section about Data set 2). Based on the data we assume the prevalence as p(t,a) = expit $\left(\beta_{0}+\beta_{1}+\beta_{2}a+\beta_{3}a^{2}\right)$. With this we calculated the maximum likelihood estimator for the coefficients $\beta_{0},\beta_{1}$,$\beta_{2}$, $\beta_{3}$. As this was a nonlinear optimization problem we solved it with the BFGS method and the initial values -2.3, 0.1, 0 and -0.001.

**Fig 3:**
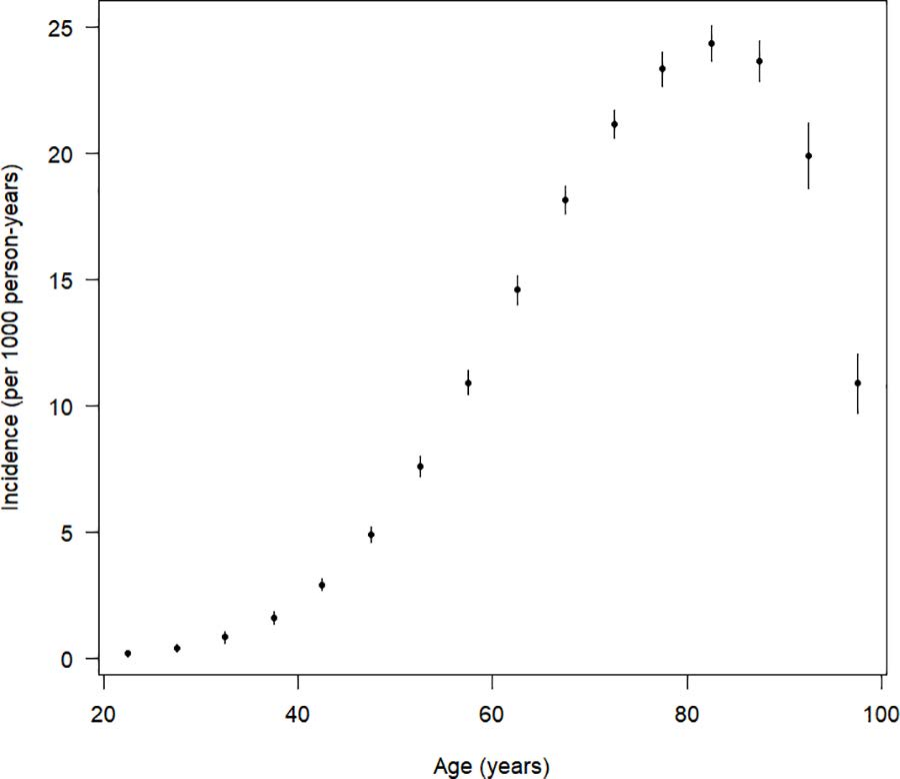
Age-specific incidence rate of diabetes in women as estimated with the plug-in estimate Eq. (9). The vertical bars indicate the 95% confidence intervals.

After the maximum likelihood estimation of p, the maximum likelihood estimate for the incidence rate *i* is calculated using the plug-in method and the delta method using the differentiable function g and its derivative $g^{\prime }$ that transforms p to i and its derivate for the calculation of the variance of i (see supplementary material). The estimates of the age-specific incidence rate including the 95% confidence intervals are shown in Fig 3.

At older ages, the length of the confidence intervals increases indicating a greater uncertainty in the estimation of the incidence rate for higher age groups.

## Discussion

We described a novel method for statistical inference with maximum likelihood estimation for the incidence rate of a chronic condition. The method is based on aggregated data and a differential equation that relates the age-specific prevalence of a chronic condition with the underlying age-specific incidence rate. With this maximum likelihood-based method the estimation of the age-specific incidence rate from age-specific prevalence data is possible. The estimator has some theoretical properties like consistency and efficiency from usage of the maximum likelihood method [11]. Additionally, it is also possible to assess the model fit, such as the expit models in the examples above, by likelihood ratio tests or with AIC/BIC that are based on the likelihood. The estimation and statistical inference of an incidence rate based on a prevalence is an epidemiologically important application. It allows usage of aggregated prevalence and mortality data instead of the conduction of longitudinal studies. So far, the estimation of the incidence rate based on the differential equation has been used in applications without a theory about statistical inference of the differential equation. Therefore, re-sampling techniques were employed to obtain confidence intervals instead. In a re-sampling procedure a number of random samples from the reported distributions of the input parameters are drawn to estimate how the uncertainty in the input parameters propagates through the differential equations into the outcomes. An example for this type of re-sampling in the field of the differential equation described above is given in [5].

The usage of the PDE and the maximum likelihood method for incidence rate estimation including corresponding confidence intervals is possible in case of non-differential as well as differential mortality. In the case of non-differential mortality, the PDE reduces to a version only depending on the incidence rate and the prevalence. Therefore, the mortality rate of diseased and non-diseased are not needed. In the case of differential mortality, it is possible to use other mortality information if information about $m_{0}$ and $m_{1}$ are missing. For a chronic condition with differential mortality, the mortality rate in the general population and the mortality rate ratio of people with the disease over people without the disease can be used for the estimation if they are known. The general mortality for these calculations may be offered from the nationwide statistical offices and mortality rate ratios could be transferred from other settings or from other countries if unknown. This can be done, because it has been shown that rate ratios provide a stable measure of association in a wide variety of human populations [20].

During the discussion of the methods described in this article, the question arises if and how it can be generalized. In the examples with differential mortality, one might consider the situation where not only the prevalence but also the information about mortality are only known with statistical uncertainty. If this is the situation, the standard error of the incidence rate requires more sophisticated concepts of error propagation, for instance influence functions [21] or Taylor series approximations of random variables [22].

A second opportunity for the generalization of the findings in this article refers to the functional form of the prevalence *p*. It is possible to have the prevalence with more information and therefore to fit more complex prevalence data. One example could be the addition of time-age-interactions. The prevalence *p*(*t,a*) can be written in terms of the expit-function *p*(*t, a*) = expit(*f*(*t, a*)) with any differentiable function *f* (for example a polynomial in *t* and *a*) in such situations. As the derivate of the expit function is given by (1 - expit) •$\begin{array}{c} \cdot \end{array}$expit and with inserting *p* = expit(*f*) into Equation (1) it immediately yields to the incidence rate estimate based on the plug-in method mentioned in the results section: *i* = *p* (∂*f* + *m*_1_ - *m*_0_) (with ∂*f* = $\frac{\partial f}{\partial t}$ + $\frac{\partial f}{\partial a}$) using this concept.

An important generalization of the method described in this article that is needed is the usability in situations with non-chronic conditions (non-zero remission rates) as we only considered diseases without remission until now. A disease where a way back from the Diseased state to Normal (see Fig 1) is possible has the remission rate *r*. Having such a disease alters the PDE in equation (1) to [23]:


$$\left(\frac{\partial }{\partial t}+\frac{\partial }{\partial a},p,=,1,-,p,i-pm_{1}-m_{0}\right)-rp.$$ (12)

In the case of non-differential mortality, the PDE in equation (12) is linear and can be solved analytically, similarly to the example about breathlessness in coal miners that was used in our analysis. If there is differential mortality with the mortality rates of diseased and non-diseased individuals being unequal (*m*_1_ ≠ *m*_0_) the analysis could be performed in a way similar to the example with type 2 diabetes in German women. However, in both cases with differential and non-differential mortality, additional information (or assumptions) about the remission rate *r* are necessary to make inference about the incidence rate *i*.

In an example from 1934 where the age-specific incidence rate of yellow fever in southern America was examined from a cross-sectional sample with data about age-specific prevalence and antibodies against yellow fever, the assumption was made that a positive serostatus does not change after an infection [24]. One could think about a similar consideration in other non-chronic conditions.

The methodology presented here has the potential for application in the context of public health and clinical questions: An example of its utility in public health can be found in the example about breathlessness. In the second example on diabetes, the methodology allows for group comparisons using the incidence rate ratio (IRR), such as comparing the diabetes incidence between people with or without inflammatory rheumatic diseases. Therefore, the methodology also has clinical applicability.

Apart from estimating incidence rates, the differential equation has been used in other applications, e.g. mortality from prevalence and incidence rate [25], in making projections about people with chronic conditions [26] or estimating the effect of health policies [27]. The maximum likelihood approach described in this paper may be advantageous in these applications, too.

This study presents a new method for incidence estimation using a maximum likelihood approach that was tested with two exemplary data sets. Further studies should analyze the statistical properties of the maximum likelihood estimators. Moreover, a simulation study should be conducted that compares the presented ML-method with other methods and examines the effectiveness of the ML-method.

In addition to that, more research about the statistical inference for the estimation of incidence rates based on the PDE that belongs to an illness-death model describing the relation between prevalence, incidence rate and mortality rates should be conducted considering methods from Bayes-statistics and MCMC.

## Supporting information

S1 Table Maximum likelihood estimators. Maximum likelihood estimators for the age-specific incidence rate of diabetes in women as estimated with the plug-in estimate Eq. (9) including 95% confidence intervals. All values are rounded to three decimal places.
(DOCX)

S1 File Supporting Information: Calculations. **Data availability statement** The source code for use with the open source statistical software R (including data and analysis) is available in the free online repository Zenodo with the following link: https://zenodo.org/records/8383574 (DOI 10.5281/zenodo.8383573); All data used is aggregated data from public sources that were cited.
(DOCX)
