# Supplementary material for: Maximum likelihood estimation of age-specific incidence rate from prevalence
Source: PLoS One. 2025 May 14;20(5):e0321924. doi: 10.1371/journal.pone.0321924 (PMC12077784; doi:10.1371/journal.pone.0321924)
Supplement: S1 Table — Maximum likelihood estimators for the age-specific incidence rate of diabetes in women as estimated with the plug-in estimate Eq. (9) including 95% confidence intervals. All values are rounded to three decimal places. (DOCX) [file pone.0321924.s001.docx]

**Title: Maximum likelihood estimators**

**S1 Table: Maximum likelihood estimators for the age-specific incidence rate of diabetes in women as estimated with the plug-in estimate Eq. (9) including 95% confidence intervals. All values are rounded to three decimal places.**

| **Middle age in age group *k* (in years)** | **ML estimator of incidence** | **95%- confidence interval of the incidence rate** |
| --- | --- | --- |
| **22.5** | 0.000 | 0.000 to 0.000 |
| **27.5** | 0.000 | 0.000 to 0.001 |
| **32.5** | 0.001 | 0.001 to 0.001 |
| **37.5** | 0.002 | 0.001 to 0.002 |
| **42.5** | 0.003 | 0.003 to 0.003 |
| **47.5** | 0.005 | 0.005 to 0.005 |
| **52.5** | 0.008 | 0.007 to 0.008 |
| **57.5** | 0.011 | 0.010 to 0.011 |
| **62.5** | 0.015 | 0.014 to 0.015 |
| **67.5** | 0.018 | 0.018 to 0.019 |
| **72.5** | 0.021 | 0.021 to 0.022 |
| **77.5** | 0.024 | 0.023 to 0.024 |
| **82.5** | 0.024 | 0.024 to 0.025 |
| **87.5** | 0.024 | 0.023 to 0.024 |
| **92.5** | 0.012 | 0.019 to 0.021 |
| **97.5** | 0.011 | 0.010 to 0.012 |
