# Supplementary material for: Maximum likelihood estimation of age-specific incidence rate from prevalence
Source: PLoS One. 2025 May 14;20(5):e0321924. doi: 10.1371/journal.pone.0321924 (PMC12077784; doi:10.1371/journal.pone.0321924)
Supplement: S1 File — Data availability statement The source code for use with the open source statistical software R (including data and analysis) is available in the free online repository Zenodo with the following link: https://zenodo.org/records/8383574 (DOI 10.5281/zenodo.8383573); All data used is aggregated data from public sources that were cited. (DOCX) [file pone.0321924.s002.docx]

**Supporting Information: Calculations**

**Illness death model**

According to the law of total probability, general mortality $m$ is a convex combination of the mortality of the diseased ($m$_1_) and healthy ($m$_0_), i.e., $m=\left( 1-p \right)m_{0}+pm_{1}$. We can solve this equation for $m_{0}$ in order to be able to insert this in the ODE in the case that general mortality and mortality of diseased are known instead of mortality of non-diseased.

$$m=\left( 1-p \right)m_{0}+pm_{1}$$

$\Leftrightarrow{- pm}_{1}+m=(1-p)m_{0}$

$\Leftrightarrow m_{0}= \frac{m-{p\cdot m}_{1}}{1-p}$

**Calculations in the case of non-differential mortality**

Having $i\left( a \right)=$exp($\gamma_{0}$+$\gamma_{1}a$) (*) with $\gamma_{0}$ and $\gamma_{1}$as coeffcients. The substitution of (*) into Equation (6) from the main text and using the initial conditions $a_{0}=20$ and $p(20)=0$ we get:

$p\left( a \right)=1-(1-0)$ exp$\left( -\int_{20}^{a} i\left( \tau\right)d\tau\right)$ with $i\left( \tau\right)$ from (*)

$=1-(1-0)$ exp$\left( -(I\left( a \right)-I\left( 20 \right)) \right)$ with $I\left( \tau\right)$ as the integral of $i\left( \tau\right)$

- $I\left( \tau\right)=\frac{exp(\gamma_{0}+\gamma_{1}\tau)}{\gamma_{1}}$ as $I'\left( \tau\right)=\gamma_{1}\cdot\frac{exp(\gamma_{0}+\gamma_{1}\tau)}{\gamma_{1}}$ = $exp(\gamma_{0}+\gamma_{1}\tau)$

$=1-1\cdot$ exp$\left( -\left( \frac{exp(\gamma_{0}+\gamma_{1}a)}{\gamma_{1}}-\frac{exp(\gamma_{0}+\gamma_{1}20)}{\gamma_{1}} \right) \right)$

$=1-$exp$\left( \frac{exp(\gamma_{0}+\gamma_{1}20)}{\gamma_{1}}-\frac{exp(\gamma_{0}+\gamma_{1}a)}{\gamma_{1}} \right)$

Using the auxiliary function $h\left( z \right)= \frac{exp(\gamma_{0}+\gamma_{1}z)}{\gamma_{1}}$ we obtain $\boldsymbol{p}\left( \boldsymbol{a} \right)\mathbf{=1-}$**exp**$\left( \boldsymbol{h(20)-h(a)} \right)$

**Calculations in the case of differential mortality**

**General mortality and mortality rate of diseased**

Substitution of $i(a)$ = exp($\gamma_{0}+\gamma_{1}a$) in equation (9)

$$G\left( a \right)= \frac{\exp\left( \gamma_{0}+\gamma_{1}a \right)-exp(\gamma_{0}+\gamma_{1}a_{0})}{\gamma_{1}+M_{1}\left( a \right)-M(a)}$$

With

$$M_{1}\left( a \right)=\frac{exp(-6.295+0.052\cdot a)}{0.052}$$

and

$$M\left( a \right)= \frac{exp(-9.300+0.092\cdot a)}{0.092}$$

**General mortality and mortality rate ratio**

The differentiable function $g$ and its derivative $g'$ that transforms $p$ to $i$ and its derivate for the calculation of the variance of $i$

$$g\left( a \right)=p\left( {\hat{\beta}_{1}}_{ML}+{\hat{\beta}_{2}}_{ML}+2a{\hat{\beta}_{3}}_{ML} \right)+\frac{m\cdot p\cdot R}{1+p\cdot R}$$

$$g'\left( a \right)=\left( {\hat{\beta}_{1}}_{ML}+{\hat{\beta}_{2}}_{ML}+2a{\hat{\beta}_{3}}_{ML} \right)+\frac{m\cdot R}{{(1+p\cdot R)}^{2}}$$

Supplementary Table 1 shows the resulting 95%- confidence intervals for the incidence rates in the example with women with type 2 diabetes.
